# Connectivity-based Cortical Parcellation via Contrastive Learning on Spatial-Graph Convolution

**DOI:** 10.34133/2022/9814824

**Published:** 2022-03-08

**Authors:** Peiting You, Xiang Li, Fan Zhang, Quanzheng Li

**Affiliations:** ^1^Massachusetts General Hospital and Harvard Medical School, Boston, MA, USA; ^2^Beijing International Center for Mathematical Research (BICMR), Peking University, Beijing, China; ^3^Brigham and Women’s Hospital and Harvard Medical School, Boston, MA, USA

## Abstract

*Objective*. Objective of this work is the development and evaluation of a cortical parcellation framework based on tractography-derived brain structural connectivity. *Impact Statement*. The proposed framework utilizes novel spatial-graph representation learning methods for solving the task of cortical parcellation, an important medical image analysis and neuroscientific problem. *Introduction*. The concept of “connectional fingerprint” has motivated many investigations on the connectivity-based cortical parcellation, especially with the technical advancement of diffusion imaging. Previous studies on multiple brain regions have been conducted with promising results. However, performance and applicability of these models are limited by the relatively simple computational scheme and the lack of effective representation of brain imaging data. *Methods*. We propose the Spatial-graph Convolution Parcellation (SGCP) framework, a two-stage deep learning-based modeling for the graph representation brain imaging. In the first stage, SGCP learns an effective embedding of the input data through a self-supervised contrastive learning scheme with the backbone encoder of a spatial-graph convolution network. In the second stage, SGCP learns a supervised classifier to perform voxel-wise classification for parcellating the desired brain region. *Results*. SGCP is evaluated on the parcellation task for 5 brain regions in a 15-subject DWI dataset. Performance comparisons between SGCP, traditional parcellation methods, and other deep learning-based methods show that SGCP can achieve superior performance in all the cases. *Conclusion*. Consistent good performance of the proposed SGCP framework indicates its potential to be used as a general solution for investigating the regional/subregional composition of human brain based on one or more connectivity measurements.

## 1. Introduction

Cortical parcellation of human brain aims to identify spatially contiguous area in cortical region, which can be characterized by distinct functional, structural, anatomical, cytoarchitectural, or genetic patterns [1]. Accurate parcellation of the cortical surface provides an essential basis for investigating brain cognitive process (e.g., in functional localization study), morphology (e.g., in developmental neuroscience study), and brain connectomics. In the works by Passingham et al. [2], it was proposed that each cortical area can be characterized by a unique pattern of inputs and outputs (“connectional fingerprint”), together with the local infrastructure characterized by the microstructural properties; these patterns can be major determinant for the function of that area. Based on the premise of the connectional fingerprint, it has been reported that voxels belonging to the same brain region usually share similar structural connectivity patterns. For example, Johansen-Berg et al. identified the border between the supplementary motor area (SMA) and pre-SMA by locating an abrupt change in their connectivity patterns [3].

Recent advancement in the imaging technology such as the diffusion-weighted magnetic resonance imaging (DWI) has enabled us for high-resolution high-quality tractography for the white matter tracts and the corresponding structural connectivity [4]. Many studies have been conducted on the feasibility for computer-assisted cortical parcellation based on structural connectivity derived from DWI images, including the parcellation for inferior parietal cortex complex [5], the lateral parietal cortex [6], and the temporoparietal junction area [7]. Most of these studies utilized unsupervised approach, such as K-means and hierarchical clustering methods, for discriminating voxels with different structural connectivity patterns. Thus, their results rely on human interpretation for identifying the desired brain region(s), usually focused on a specific area. For the supervised learning scheme, there is generally a lack of brain imaging data with detailed voxel-wise labeling. Further, direct mapping from the connectivity pattern to the voxel label can only be trained on a specific region, which limits the applicability of the trained model [8, 9]. In addition, rather than representing brain imaging data in the volumetric Euclidean space or as independent feature vectors, increasing number of studies has recognized the importance of utilizing graph theory [10] or performing the image analysis on the graph [11].

To address the above challenges, we have developed the Spatial-graph Convolution Parcellation (SGCP) framework for learning the spatial-graph representation from the input structural connectivity data and performing cortical parcellation via a two-stage contrastive learning scheme. SCGP overcomes the need for extensive and accurate voxel labels by a self-supervised contrastive learning scheme and the graph augmentation techniques, which have been widely used in various computer vision tasks including medical image analysis [12–14]. It employs a graph convolution network- (GCN-) based method for encoding the structural connectivity patterns. GCN leverages the powerful representation learning capability of layered convolution filtering as used in convolution neural network (CNN) [15], while performing the convolution analysis on a graph rather than on the Euclidean space [16]. Thus, GCN is more feasible and effective for analyzing data intrinsically reside on a graph-defined manifold such as social network data for recommendation system [17], medicinal chemistry data for drug discovery [18], as well as brain imaging data where voxels are governed by the underlying brain network (s) [19, 20]. SGCP also features a spatial-graph convolution network (SGCN) filter design, so that both geometric and topological information of the voxels can be used together, which will lead to more spatially consistent parcellation results. Performance comparison of SGCP with traditional machine learning-based methods and other graph-based deep learning methods on the public Human Connectome Project (HCP) data shows that the proposed framework can achieve superior parcellation accuracy with consistent spatial and connectivity patterns of the parcellated results. Source code of this work can be found in https://github.com/rachelyou/CL-SGCN.

## 2. Results and Discussion

### 2.1. Performance of the Region Parcellation Task

Based on the binary voxel-wise classification results, we use the Dice score to measure the similarity between the regions defined by parcellation results and the regions defined in the DK atlas (regarded as ground truth), which are listed in Table 1. A higher Dice score indicates that the two regions are spatially more similar to each other, ranging from 0~1.

Table 1Performance measured by Dice score between the parcellated regions and DK Atlas regions (regarded as ground truth). Top: results from left hemisphere; Bottom: results from right hemisphere.

**(a):** 

Subjects	1	2	3	4	5	6	7	8	9	10	11	12	13	14	15
PC.L	0.82	0.87	0.84	0.88	0.81	0.84	0.87	0.87	0.87	0.88	0.86	0.88	0.84	0.83	0.85
LO.L	0.89	0.89	0.88	0.90	0.90	0.87	0.89	0.92	0.92	0.88	0.87	0.91	0.83	0.84	0.82
InP.L	0.90	0.86	0.91	0.88	0.88	0.88	0.89	0.91	0.89	0.89	0.86	0.88	0.86	0.90	0.85
EC.L	0.84	0.86	0.81	0.83	0.85	0.82	0.81	0.86	0.80	0.85	0.80	0.87	0.83	0.83	0.83
RMF.L	0.91	0.89	0.89	0.90	0.89	0.86	0.92	0.90	0.89	0.86	0.91	0.87	0.88	0.90	0.88

**(b):** 

Subjects	1	2	3	4	5	6	7	8	9	10	11	12	13	14	15
PC.R	0.83	0.89	0.82	0.84	0.83	0.88	0.87	0.88	0.88	0.89	0.90	0.90	0.86	0.80	0.88
LO.R	0.88	0.91	0.85	0.89	0.88	0.90	0.90	0.83	0.84	0.86	0.87	0.88	0.80	0.87	0.90
InP.R	0.89	0.86	0.89	0.90	0.89	0.92	0.92	0.90	0.91	0.91	0.88	0.91	0.87	0.86	0.87
EC.R	0.83	0.88	0.87	0.80	0.86	0.86	0.86	0.87	0.87	0.84	0.83	0.83	0.85	0.85	0.84
RMF.R	0.90	0.89	0.82	0.90	0.91	0.89	0.91	0.91	0.90	0.87	0.91	0.87	0.89	0.88	0.85

**Figure 1:**
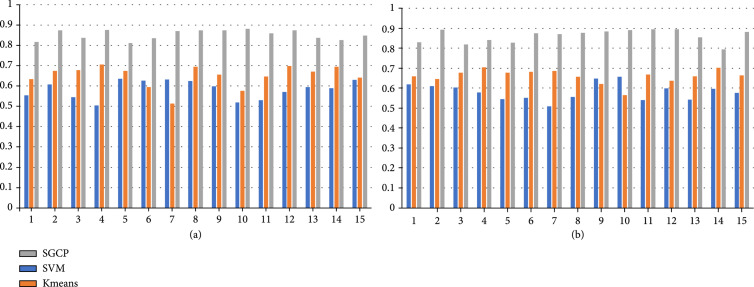
Performance comparison between SGCP, SVM, and K-means based on the Dice score for parcellating the precentral gyrus region. Indices of the 15 subjects are listed in the x-axis, and the Dice scores are shown in the y-axis. (a) left hemisphere and (b) right hemisphere.

Following the similar methodology designs in the previous works of connectivity-based parcellation [6, 7, 21, 22], we have implemented support vector machine (SVM) and K-means algorithm to perform the same task of parcellating the five brain regions as listed above. For the K-means algorithm, the individual cross-correlation matrix is used as the input to group voxels with similar connectivity profiles together. For the SVM algorithm, connectivity profiles are used as input to perform voxel-wise classification. Performance comparison between SGCP and two baseline methods (SVM and K-means) on the task of parcellating precentral gyrus (PC) for all the subjects is shown in Figure 1 as an example, and the averaged Dice scores for parcellating all five regions are listed in Table 2.

**Table 2:** Performance comparison between SGCP, SVM, and K-means based on the averaged dice score across 15 subjects, on the parcellation of five regions (left and right) in this study.

	PC.L	LO.L	InP.L	EC.L	RMF.L	PC.R	LO.R	InP.R	EC.R	RMF.R
SVM	0.59	0.64	0.65	0.64	0.57	0.58	0.65	0.65	0.65	0.56
K-means	0.65	0.62	0.60	0.62	0.67	0.66	0.59	0.60	0.62	0.66
SGCP	0.85	0.88	0.88	0.83	0.89	0.86	0.87	0.89	0.85	0.89

### 2.2. Performance Comparison with Baseline Methods and Ablation Study

To evaluate the effectiveness of different components in the SGCP framework, including SGCN and the contrastive learning scheme, we have implemented various baseline methods by (1) node2vec [23], which learns the feature representation of nodes in a graph based on graph characteristics and node neighborhoods. After embedding node features by node2vec, a 2-layer MLP is then trained to predict the node labels. Parameters of node2vec are set as follows: walk steps: 80, walk length:10, window size:5, and random walk probability: 0.25/4; (2) struc2vec [24], which learns the node feature representation based on graph structural similarity. Similar to node2vec, we also employ struct2vec to embed node features and train a 2-layer MLP for node label prediction. Parameters of struct2vec are set as follows: random walk length:10, the number of random walk steps:100, and window size:5; (3) substituting the core SGCN with traditional GCN, to investigate how geometric information can assist the graph feature embedding; and (4) formulating the whole framework as an end-to-end, supervised approach based on SGCN, which takes the input of the same graph representation and directly infers the voxel-level labels. In addition, we have investigated the effect of different network structures (number of layers in SGCN and the MLP classifier) on model performance. Performance comparisons are listed in Table 3, each row corresponding to a specific method or model component setting.

**Table 3:** Comparison of parcellation performance as measured by Dice score on the 5 brain regions analyzed in this work.

	PC.L	LO.L	InP.L	EC.L	RMF.L	PC.R	LO.R	InP.R	EC.R	RMF.R	Average
node2vec	0.65	0.67	0.63	0.68	0.65	0.67	0.67	0.66	0.68	0.67	0.66
struc2vec	0.64	0.65	0.60	0.65	0.66	0.65	0.66	0.64	0.65	0.66	0.64
GCN	0.65	0.70	0.67	0.71	0.67	0.70	0.69	0.69	0.73	0.68	0.69
SGCN, Supervised non-CL	0.72	0.71	0.71	0.79	0.72	0.74	0.72	0.73	0.78	0.73	0.73
SGCN (2 layers) + CL + MLP (2 layers)	0.86	**0.89**	**0.88**	0.83	**0.89**	0.88	**0.88**	**0.89**	0.85	**0.89**	**0.87**
SGCN (3 layers)+ CL + MLP (2 layers)	0.88	0.87	0.84	0.80	0.85	0.85	0.86	0.89	0.87	0.89	0.86
SGCN (2 layers) + CL + MLP (3 layers)	**0.89**	0.86	0.87	**0.84**	0.86	**0.89**	0.88	0.88	**0.87**	0.87	0.87

node2vec: unsupervised node feature embedding by node2vec, followed by a 2-layer MLP. struct2vec: unsupervised node feature embedding by strct2vec, followed by a 2-layer MLP. GCN: substituting SGCN with traditional GCN, keeping all other components as the same. SGCN, supervised non-CL: the single stage, end-to-end, supervised framework for parcellation based on SGCN. SGCN (2 layers) + CL + MLP (2 layers): the current setting used by SGCP. SGCN (3 layers) + CL + MLP (2 layers) and SGCN (2 layers) + CL + MLP (3 layers): settings where the layers in SGCN, and stage 2 MLP are increased to 3 layers, all other components are kept the same. Best parcellation performance for each region among all methods is highlighted in bold text.

### 2.3. Spatial Distribution and Structural Connectivity Patterns of the Parcellated Regions

**Figure 2:**
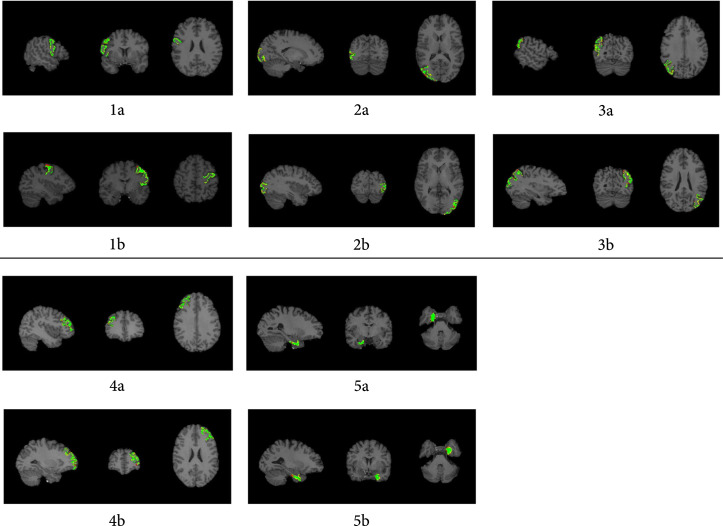
Overlay of the SGCP parcellation results combined with ground truth regions defined in DK atlas on T1w images from a random subject. Selected slices are visualized for maximized region visibility. green: overlapped voxels between the two regions; red: voxels that are presented only in the parcellated regions but not the ground truth; yellow: voxels that are presented only in the ground truth region. 1a-5a: visualizations of the PC.L, LO.L, InP.L, EC.L, and RMF.Lregions. 1b-5b: visualizations of the PC.R, LO.R, InP.R, EC.R, and RMF.R regions.

In addition to the Dice score for quantitively evaluating the performance of the proposed SGCP framework, we have also overlayed the parcellated regions with the ground truth (defined by DK atlas) onto 2D slices of the 3D volumetric brain T1w image, in order to visually check whether the parcellated brain regions are neuroscientifically meaningful. Visualizations of the overlay are shown in Figure 2.

**Figure 3:**
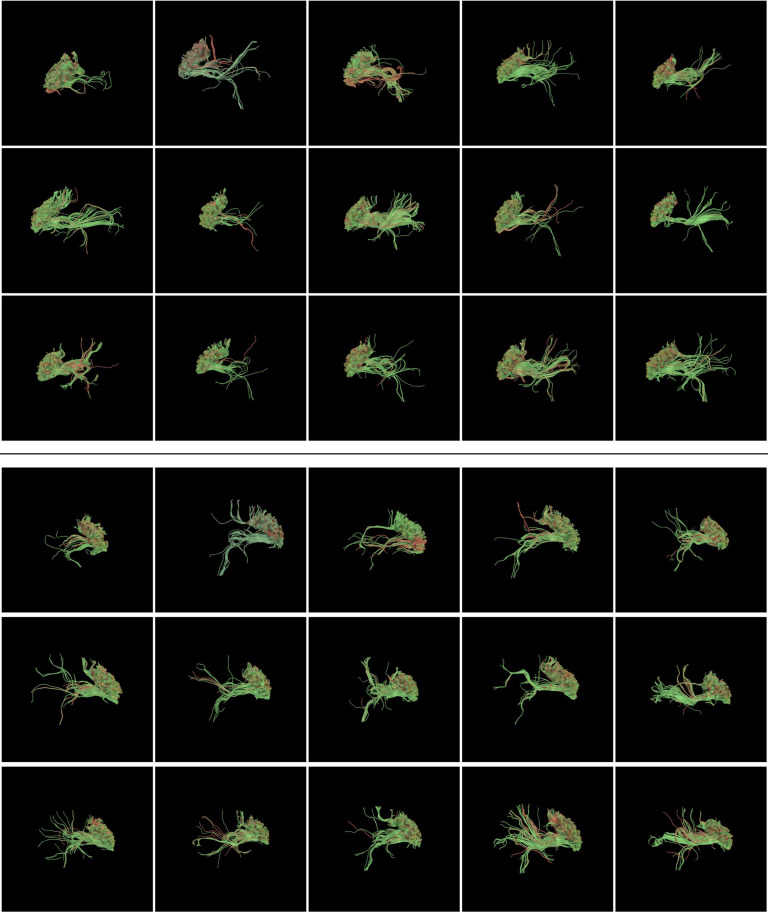
Patterns of fiber bundles connecting the parcellated regions (green) and voxels outside (red), on the PC.L (Precentral Gyrus left) region (top) and PC.R (Precentral Gyrus right) region (bottom), from the 15 subjects analysed in this study.

We have also visualized the structural connectivity patterns of the parcellated regions, as well as voxels around the parcellation results. A sample illustration of the precentral gyrus is shown in Figure 3. These visualizations represent fiber bundles connecting voxels in the voxels within(green)/outside(red) the parcellated regions to the whole brain, illustrating the differences in their connectivity patterns.

## 3. Materials and Methods

### 3.1. Study Population and Image Acquisition

We used imaging data from 15 healthy adults in the Human Connectome Project (HCP) database [37]. The HCP MRI data were acquired with a high-quality image acquisition protocol using a customized Connectome Siemens Skyra scanner. Acquisition parameters for the T1 weighted imaging (T1w) data were TE=2.14 ms, TR=2400 ms, and voxel size=0.7×0.7×0.7 mm. Acquisition parameters used for the HCP DWI data were TE=89.5 ms, TR=5520 ms, phase partial Fourier=6/8, and voxel size=1.25×1.25×1.25mm. A total of 288 volumes were acquired for each subject, including 18 baseline volumes with a low diffusion weighting b=5 s/mm and 270 volumes evenly distributed at three shells of b=1000/2000/3000 s/mm.

### 3.2. Data Preprocessing

The DWI data used in this work was processed with the well-designed HCP minimum processing pipeline [38], which includes brain masking, motion correction, eddy current correction, EPI distortion correction, and coregistration with the anatomical T1w data. Each subject’s T1w image was parcellated into 34 cortical regions of interest (ROIs) per hemisphere based on the Desikan-Killiany (DK) Atlas [39, 40]. The ROIs investigated in this study are listed in Table 4. We used the FSL tools FDT and PROBTRACTX [41] to perform probabilistic tractography based on each subject’s DWI data. For the tractography analysis, we restricted seed mask for streamline tracking in FSL to white matter voxels in a specifically predefined region (named as the “target region”). The target region covers all the voxels in the DK atlas-defined ROI to be analyzed and parcellated (e.g., the precentral gyrus), as well as voxels around this ROI to the extent of 1.5 times larger of the original ROI. We designed this “target region” scheme to test the feasibility of using structural connectivity to segment out the morphology-derived ROI from its surrounding voxels. We then set the target of the tracking to voxels in all ROIs in the DK atlas, covering the whole brain. Outputs from the tractography are two connectivity matrices, the intraregion connectivity matrix A and the interregion connectivity matrix X. Matrix A contains the voxel-voxel connection only within the target region. $A_{ij}=1$ if there is at least one tracked fiber connecting voxel i and voxel j in the target region, and $A_{ij}=0$ otherwise. Matrix X contains the voxel-region connection from each voxel within the target region to all ROIs in the DK atlas by counting the number of tracked fibers connecting each voxel to each ROI. This voxel-region connectivity density can potentially reveal the connectivity pattern difference within the target region.

Table 4Name and abbreviation of the five brain regions analysed in this study, as well as the number of nodes and edges in their corresponding graph representations.

**(a):** 

Brain region	Abbv.	# of nodes	# of edges
Precentral gyrus left	PC.L	2890	34896
Lateral occipital left	LO.L	3471	43260
Inferiorparietal left	InP.L	3815	49309
Entorhinal cortex left	EC.L	1403	26724
Rostral middle frontal left	RMF.L	3582	46977

**(b):** 

Brain region	Abbv.	# of nodes	# of edges
Precentral gyrus right	PC.R	2538	31340
Lateral occipital right	LO.R	3293	41391
Inferiorparietal right	InP.R	4366	58779
Entorhinal cortex right	EC.R	1183	20342
Rostral middle frontal right	RMF.R	3387	42881

**Figure 4:**
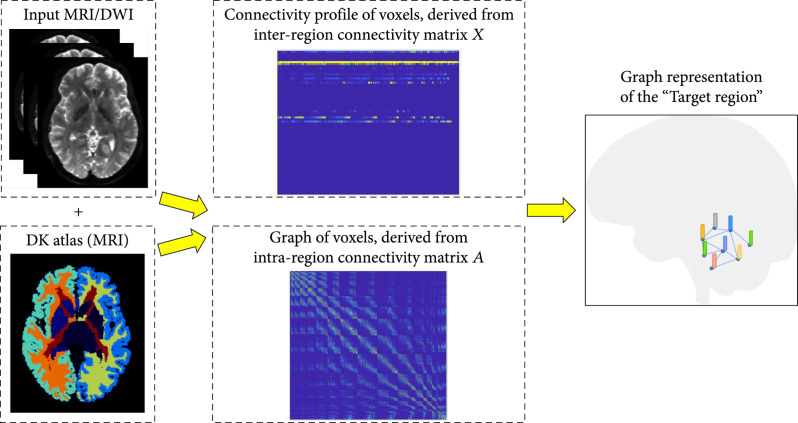
Overview of the processing pipeline. Voxels in the target region of the input DWI image are transformed into its graph representation with node features defined by the interregion connectivity matrix and graph topology defined by the intraregion connectivity matrix.

Based on the tractography results, the connectivity profile of a given voxel could be then defined as the fiber density vector ${\left(x_{1},x_{2},\cdots ,x_{n}\right)}^{T}$, where $x_{i}$ is the number of white matter fibers connecting from the given voxel to the ith ROI derived from the interregion connectivity matrix X and n is the number of regions in the DK atlas. At the same time, the topology (i.e., edges) among voxels in the target region is modeled by the graph defined by the intraregion connectivity matrix A (Figure 4).

### 3.3. Architecture Overview

**Figure 5:**
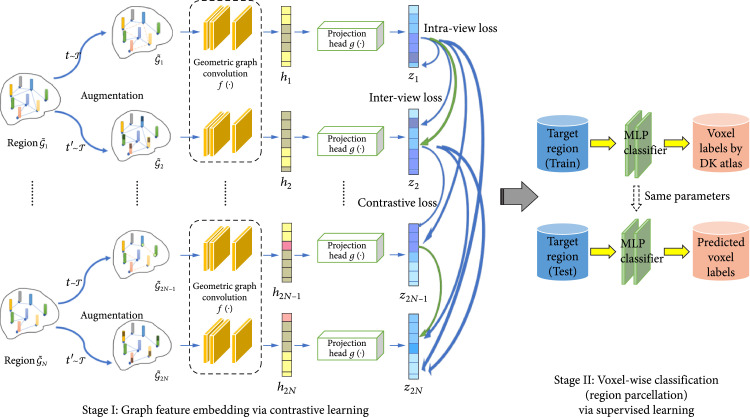
Analysis pipeline of the SGCP model. Contrastive learning-based graph feature embedding for the input image is performed at stage I (left). With the embedded features, brain region parcellation can be achieved by voxel-wise classification via supervised learning at stage II (right).

Our proposed SGCP model learns latent representations of the given graph representation from the input brain imaging data and performs voxel-wise classification for brain region parcellation, where in this study we use the DWI image and the derived structural connectivity as an example. An undirected graph $G=\left(V,E\right)$ is defined to represent the input target region, where $\;V=\left\{v_{1},v_{2},\cdots ,v_{N}\right\}$ contains the N nodes representing N voxels in the target region and E is the edge set representing the connectivity between two nodes $v_{i}$ and $v_{j}$. G has an associated node feature set $X=\left\{x_{1},x_{2},\cdots ,x_{N}\right\}$, where $x_{i}$ is the feature vector of the node $v_{i}$. As illustrated in Figure 5, SGCP is composed of two stages: the label-free, self-supervised contrastive graph feature embedding stage with geometric GCN, where positive augmented molecule graph pairs are contrasted with representations from negative pairs; and the down streamed supervised learning-based classification stage, where voxel labels (i.e., parcellated brain region) are inferred by a multilayer perceptron (MLP) based on the extracted features. In the following section, we will describe the technical components in the proposed SGCP model: the Self-Supervised Graph Contrastive Learning scheme in Section 3.4, the Graph Augmentation techniques in Section 3.5, the Spatial-graph Convolution Network in Section 3.6, and the Voxel Classification and Region Parcellation model in Section 3.7.

### 3.4. Self-Supervised Graph Contrastive Learning

Motivated by the recent development of contrastive learning in the field of machine learning and the increasing adaptation of it in computer vision, we employ a graph contrastive learning framework similar with works in [14] for self-supervised graph embedding of the input data. The framework follows the common graph contrastive learning paradigm, which aims to learn an effective representation that can maximize the interaction between different views of the data. As shown in Figure 5, graph augmentations are performed on the input data (i.e., graph representation of the target region G) to generate different views of G. Detailed specifications of the augmentation technique will be provided in the section 3.5. Then, a contrastive objective is used to enforce the embedding of each node in the two views to be consistent with each other and at the same time can be distinguished from the embedding of other nodes [42]. Specifically, denote T as the set of arbitrary augmentation functions. Without loss of generality, here, we use two augmentation functions, where $t,t^{\prime }\sim T$ are two different augmentation functions independently sampled from T. These two graph views are then generated by applying the different augmentation functions on the same graph, denoted as ${\tilde{G}}_{1}=t\left(\tilde{G}\right)$ and ${\tilde{G}}_{2}=t^{\prime }\left(\tilde{G}\right)$. An encoder function $f\left(∙\right)$, which can be implemented by any transform function, then embeds features on the nodes with attributes from all the augmented graph samples: $h_{1}=f\left({\tilde{X}}_{1},{\tilde{A}}_{1}\right)$ and $h_{2}=f\left({\tilde{X}}_{2},{\tilde{A}}_{2}\right),$ where ${\tilde{X}}_{*}$ and ${\tilde{A}}_{*}$ are the feature matrix and adjacency matrix of the generated graphs ${\tilde{G}}_{1}$ and ${\tilde{G}}_{2}$, respectively, and $h_{*}$ is the embedded output of the encoder. While most of the recent works employed GCN-like networks [14] as the encoder function $f\left(∙\right)$, in this work in order to leverage the spatial relationship among the graph nodes (which are voxels in Euclidean space), we will use the spatial-graph convolution network (SGCN) as the encoder, which will be described in section 3.6. After obtaining all the graph feature embeddings of $h_{*}$, they will be fed into a projection head $g\left(∙\right)$ implemented by a small multilayer perceptron (MLP) to obtain a metric embedding $z_{1}=g\left(h_{1}\right)$, $z_{2}=g\left(h_{2}\right)$, where $z_{1},z_{2}\in {\mathbb{R}}^{d\prime }$ with $d^{\prime }<d$, which is in a lower dimensional space compared with the dimension of $h_{*}$.

After the setting up of graph feature and metric embedding, parameters of the encoder $f\left(∙\right)$ and nonlinear projection head $g\left(∙\right)$ will be optimized by the contrastive objective, which encourages the distance between the metric embedding of the same node in the two different views to be small, and the distance between the metric embeddings with other nodes to be large. Specifically, for a given node $v_{i}$, its embedding generated in one view $z_{1}^{i}$ and in the other view $z_{2}^{i}$ will form the positive pairs. Embeddings of the other node $v_{*}$ in the two views are naturally regarded as the negative pairs. Based on the nonparametric classification loss InfoNCE [43], multiview graph contrastive learning loss [42] can be defined for each positive pair $\left(z_{1}^{i},z_{2}^{i}\right)$ as

(1)lz1i,z2i=logeθz1i,z2i/τeθz1i,z2i/τ+∑k≠ieθz1i,z2k/τ+∑k≠ieθz1i,z1k/τ.

τ is the hyperparameter that controls the sensitivity of the embedding. $\theta \left(∙\right)$ measures the similarity between two embeddings, here, we use the cosine similarity function $s\left(∙,∙\right)$ to define $\theta \left(z_{1},z_{2}\right)=s\left(g\left(z_{1}\right),g\left(z_{2}\right)\right)$. In Equation (1), the second and the third term in the denominator calculates similarities between negative pairs from interview and intraview nodes, respectively. The overall objective L to be optimized is then defined as the average over all positive pairs

(2)L=12N∑i=1Nlz1i,z2i+lz2i,z1i.

### 3.5. Graph Augmentation

In machine learning, data augmentation is the commonly-used method for creating a comprehensive set of possible data points, thus enhancing the model generalizability and robustness [44]. In the context of self-supervised learning, such as contrastive learning, the data augmentation is even more important for generating data needed for training the model without relying on data labels. In the works of [14, 42], various graph-based augmentation techniques have been proposed, such as node dropping, edge deletion, subgraph, and feature masking. In this work, we will employ the techniques of edge deletion and feature masking to constitute the augmentation functions set T. Graph views ${\tilde{G}}_{1}$, ${\tilde{G}}_{2}$ can then be generated by jointly performing the two graph augmentation techniques on the given graph G.

*Edge deletion*: in this augmentation process, we will randomly remove edges in the graph based on a predefined edge importance to generate semantic-consistent views of the graph. Given a node degree centrality measure: $\varphi_{c}\left(∙\right)\text{: }V⟶{\mathbb{R}}^{+}$, we can define edge centrality as the average of the centrality score of the two nodes connected, $w_{uv}^{2}=\left(\varphi_{c}\left(u\right)+\varphi_{c}\left(v\right)\right)/2$. Based on the edge centrality, importance of the edge connecting node u,v can defined as follows, following the same method as introduced in [42]:

(3)puve=1−minsmaxe−suvesmaxe−μse∙pe,pτ,

where $s_{uv}^{e}=\log w_{uv}^{e}$ to alleviate the impact from densely connected nodes, $p_{e}$ is the hyperparameter controlling the overall probability of removing edges, $s_{\max}^{e}$ and $\mu_{s}^{e}$ is the maximum and average of the centrality of all edges, and $p_{\tau }$ is a cut-off probability to avoid overly-corrupting the graph. Then, we will delete edges from the given graph with a probability of $p_{uv}^{e}$, with the premise that more important edge (as characterized by $p_{uv}^{e}$) shall be less likely to be deleted in order to preserve the graph semantics.

*Feature masking*: in this augmentation process, we will randomly mask-out node features based on feature importance. Specifically, for node u in the graph, importance of its ith feature [42] can be calculated as

(4)wif=∑u∈Vxui∙φcu,

where $\varphi_{c}\left(∙\right)$ is the node degree centrality which reflects the node importance, and $x_{ui}\in \left\{0,1\right\}$ measures the occurrence of the ith feature in node u. Similar to the edge deletion process, the probably of masking-out the ith feature in node u is

(5)pif=1−minsmaxf−sifsmaxf−μsf∙pf,pτ,

where $s_{i}^{f}=\log w_{i}^{f}$ following the similar purpose of alleviating the impact from densely connected nodes, $s_{\max}^{f}$ and $\mu_{s}^{f}$ is the maximum and average value of $s_{i}^{f}$, respectively, and $p_{f}$ is a hyperparameter that controls the overall level of feature masking probability.

### 3.6. Spatial-Graph Convolution Network

Graph representation of nonEuclidean data has been widely investigated in various fields, with the adoption of graph convolution network-based frameworks [16]. In the brain imaging analysis, we have seen increasing studies utilizing GCNs for performing the functional [45], pathological [20], and multimodal modeling of the brain [46]. Most of the current GCN frameworks utilize a neighborhood node aggregation operation, conceptually similar to the pooling operation in CNNs, which iteratively updates the node features [16]. Various node aggregation strategies have been proposed to improve the performance of GCNs, including the introduction of attention mechanism [47] and the structured aggregation [48]. One unique characteristics of the volumetric brain imaging analysis as in this work is that nodes are defined both on the graph (i.e., underlying brain networks) and the Euclidean space (as nodes are essentially voxels in the 3D image). Thus, we will utilize spatial-GCN (SGCN) [49] as the core graph encoder function $f\left(∙\right)$ for the proposed SGCP framework, as traditional GCNs do not use the spatial (geometric) information of the nodes. Unlike other node aggregation schemes, SGCN performs node aggregation based on both graph topology and the spatial position among the nodes, to leverage information over the geometric structure of the image. For a given graph $G=\left(V,E\right)$, let $H=\left[h_{1},h_{2},\cdots ,h_{n}\right]$ denote the matrix of node features to be filtered by the convolution layer. $h_{i}\in {\mathbb{R}}^{d_{\text{in}}}$ are the column vectors, where the dimension $d_{\text{in}}$ is determined by the number of filters in the previous layer. In addition, we have coordinate $p_{i}\in {\mathbb{R}}^{t}$ for node $v_{i}$, which is constant across layers as they are the intrinsic property of the nodes. The spatial-graph aggregation operation can then be defined on node $v_{i}$, based on both the coordinate information $p_{i}$ and the graph neighborhood information $N_{i}=\left\{j:e_{ij}=1\right\}$:

(6)h¯iU,b=∑j∈NiReLUUTpj−pi+b⨀hj,

where $U\in {\mathbb{R}}^{t\times d}$, $b\in {\mathbb{R}}^{d}$ are trainable parameters, d is the dimension of $h_{j}$, ⨀ is element-wise multiplication, and ${\bar{h}}_{i}$ is the feature representation of node $v_{i}$ after the convolution operation. It can be seen that spatial positions of node $v_{i}$ and its adjacent nodes are transformed using a linear operation combined with nonlinear ReLU function. Convolution operations with spatial-graph aggregation can be easily extended to multiple filters with a set of spatial aggregation parameter U and b for each filter:

(7)h¯iU,B=h¯iU1,b1⊕⋯⊕h¯iUk,bk,

where ⨁ denotes the vector concatenation.

### 3.7. Voxel Classification and Region Parcellation

As the contrastive learning-based graph embedding scheme in Stage I of the SGCP model is label-free, in order to perform cortical parcellation of the given target region, we will train a supervised classification model implemented by a 3-layer MLP for voxel classification, using the embedded graph features on each node/voxel as input. Recalling that the “target region” in this work is defined by the region containing both voxels within the brain region defined by the DK atlas (e.g., the precentral gyrus) and the voxels outside extending to 1.5 times larger of that region; thus, for each target region, we will have the voxel label of “1” if it belongs to the part of the parcellated brain region or label of “0” if it is outside the parcellated brain region. In this way, we can parcellate the desired brain region from the target region based on the predicted voxel labels. As totally five regions are analyzed in this work (EC, PC, RMF, InP, and LO), we design the cross-validation scheme of training the classifier with voxels belonging to four regions and then test the classifier on the left-out region. For example, to evaluate the parcellation performance of SGCP on EC, we will train the classifier on voxels and their corresponding graph feature embeddings in the four target regions defined on PC, RMF, InP, and LO and then test it on the voxels in target region defined on EC. It should be noted that the graph feature embeddings which are derived from the contrastive learning framework in Stage I are kept constant in Stage II; thus, in each folds of cross-validation, only a new MLP needs to be retrained.

## 4. Conclusion and Discussion

In this study, we design and implement the spatial-graph convolution parcellation (SGCP) framework based on a contrastive learning scheme and spatial-graph representation modeling. The proposed framework is evaluated on 5 brain regions from 15 subjects based on the Dice score between the parcellation results and the ground truth regions defined by the DK atlas. As SGCP has shown consistent performance of Dice score>0.8 over all the 15 subjects and all the 5 regions (Table 1), it has the potential to be used as a tool for analyzing the structural and functional delineations of the brain regions and their subregions. Comparison with traditional methods for connectivity-based cortical parcellation shows that SGCP can achieve much superior performance (Figure 1 and Table 2).

From the ablation study (Table 3), we can observe that (1) substituting SGCN used in the proposed SGCP framework with traditional GCN, which causes the node feature aggregation no longer leveraging spatial information, will severely decrease the parcellation performance. Geometric relationship among nodes (voxels) is particularly important for the task of parcellation, as the ground truth brain region is generally defined as a congregated 3D shape that is spatially continuous. (2) Contrastive learning scheme is also very important for the parcellation task, as directly performing parcellation (second row in Table 3) results in much lowered accuracy. This could be caused by the fact that structural connectivity patterns in the 5 regions studied in this work are not the same, thus cannot be characterized by a simple supervised scheme. (3) Performance of the SGCP framework is not sensitive to the configuration of network structure for both the encoder network (SGCN) and the classification network.

Examination on the spatial distribution of the parcellated brain regions (Figure 2) confirms that SGCP results are spatially consistent with the ground truth regions, as most of the voxels are overlapping (colored in green). We have observed slightly missing voxels near the cortical surface in the parcellated results (colored in red, indicating these voxels are only presented in the ground truth regions), which can be due to the increasing fiber crossings near the cortical surface [25], also cognized as the “superficial white matter systems” where the complex arrangement of white matter fibers residing just under the cortical sheet [26], and consequently the difficulty in performing the correct fiber tracking. Fiber bundles connecting the parcellated regions (visualized as green polylines in Figure 3) show very consistent connectivity pattern, with distinct connectivity patterns of the voxels outside the parcellated regions (visualized as red polylines in Figure 3).

While the proposed two-phase SGCP framework outperformed direct supervised learning-based GCN as shown in Table 3, there exists improved supervised contrastive learning frameworks such as the SupCon method proposed in [27]. By formulating the contrastive loss with considerations both from augmented graph (self-supervised) and nodes with the same class labels (supervised), SupCon can achieve superior performance compared with traditional contrastive learning models such as SimCLR [28]. In our future works, we will also explore the feasibility of utilizing a similar strategy to merge the node feature embedding phase with the node classification phase to achieve end-to-end parcellation.

In addition to the volumetric parcellation (i.e., each graph node is a 3D voxel) as proposed in this work, there exists other studies performing parcellation based on different representations of the brain. For example, works of Ge et al. [29] parcellated brain region of interests (ROIs) based on predefined atlas into multiscale subnetworks. Works by Cucurull et al. [30] reconstructed the cortical surface into its graph representation where each node represents a vertex of the surface mesh then utilized GCN to parcellate the cortical surface into different brain areas. Works by Liu et al. [31] utilized GCN to parcellate fiber bundles, where graph nodes were uniformly-sampled points along the fiber tracts, and the graph edges were the geometric relationships among sampling points. As the SGCP is a general graph analytics framework and not limited to a specific type of data (volume, ROI, mesh surface, or fiber bundle), we can potentially applied SGCP to these data types as well.

Currently, SGCP performs parcellation on a predefined “target region” which is a spatial extension of the ground truth region. In practice, without the knowledge of the ground truth region definition, we can apply SGCP on a manually defined region of interest with arbitrary shapes (e.g., a rectangular box or a sphere). We are also exploring the individualized, whole-brain, voxel-wise parcellation by SGCP with the assistance of a global brain atlas, while tackling the technical challenge of memory limitation and computational cost. Alternatively, we can also try the iteratively, hierarchical parcellation of the brain, inspired by the works of [29]. More importantly, as the major modeling of SGCP is formulated in a self-supervised scheme, we are testing its capability to perform subregion parcellation, investigating the unique structural-functional characteristics of the fine-grained compositions in certain brain regions, such as the entorhinal cortex, where preliminary studies have shown the presence of subregion with distinguished connectivity patterns in different cortical pathways [32, 33]. Finally, while in this study SGCP is used to analyze structural connectivity patterns derived from DWI images, it can be applied to functional connectivity derived from fMRI or MEG/EEG data [34, 35], formulating a structural-functional parcellation framework [36]. Further, rich information can be encoded in the node features, including morphological features derived from T1 imaging, pathological and proteinopathies features derived from PET imaging, as well as genetic features derived from microarrays.

## Data Availability

The DWI and T1w MRI data that support the findings of this study are available at the Human Connectome Project, http://www.humanconnectomeproject.org/. IDs of the 15 subjects used in this study are 100307, 100408, 101107, 101309, 101915, 103111, 103414, 103818, 105014, 105115, 106016, 108828, 110411, 111312, and 111716.
